# Supplementary material for: ATM inhibition exploits checkpoint defects and ATM-dependent double strand break repair in TP53-mutant glioblastoma
Source: Nat Commun. 2024 Jun 21;15:5294. doi: 10.1038/s41467-024-49316-8 (PMC11192742; doi:10.1038/s41467-024-49316-8)
Supplement: Supplementary file 1 — Supplementary Information [file 41467_2024_49316_MOESM1_ESM.docx]

**Supplementary Information**

**ATM Inhibition Exploits Checkpoint Defects and ATM-Dependent Double Strand Break Repair in *TP53*-Mutant Glioblastoma**

Daniel J. Laverty^1^; Shiv K. Gupta^2^; Gary A. Bradshaw^3^; Alexander S Hunter^3^; Brett L. Carlson^2^; Nery Matias Calmo^1^; Jiajia Chen^2^; Shulan Tian^2^; Jann N. Sarkaria^2^; Zachary D. Nagel^1^

1. Harvard T.H. Chan School of Public Health, Boston, MA 02115
2. Mayo Clinic, Rochester, MN 55905
3. Harvard Medical School, Boston, MA, 02115

Corresponding author: Zachary D. Nagel [znagel@hsph.harvard.edu](mailto:znagel@hsph.harvard.edu)

Contents:

Supplementary Figures S1-S20

Supplementary Table S1 and S2

Supplementary References

**
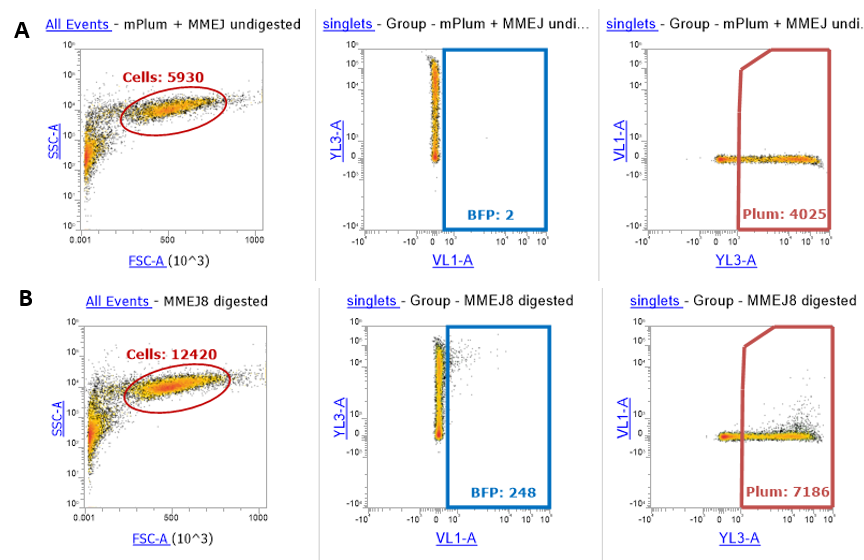
**

**
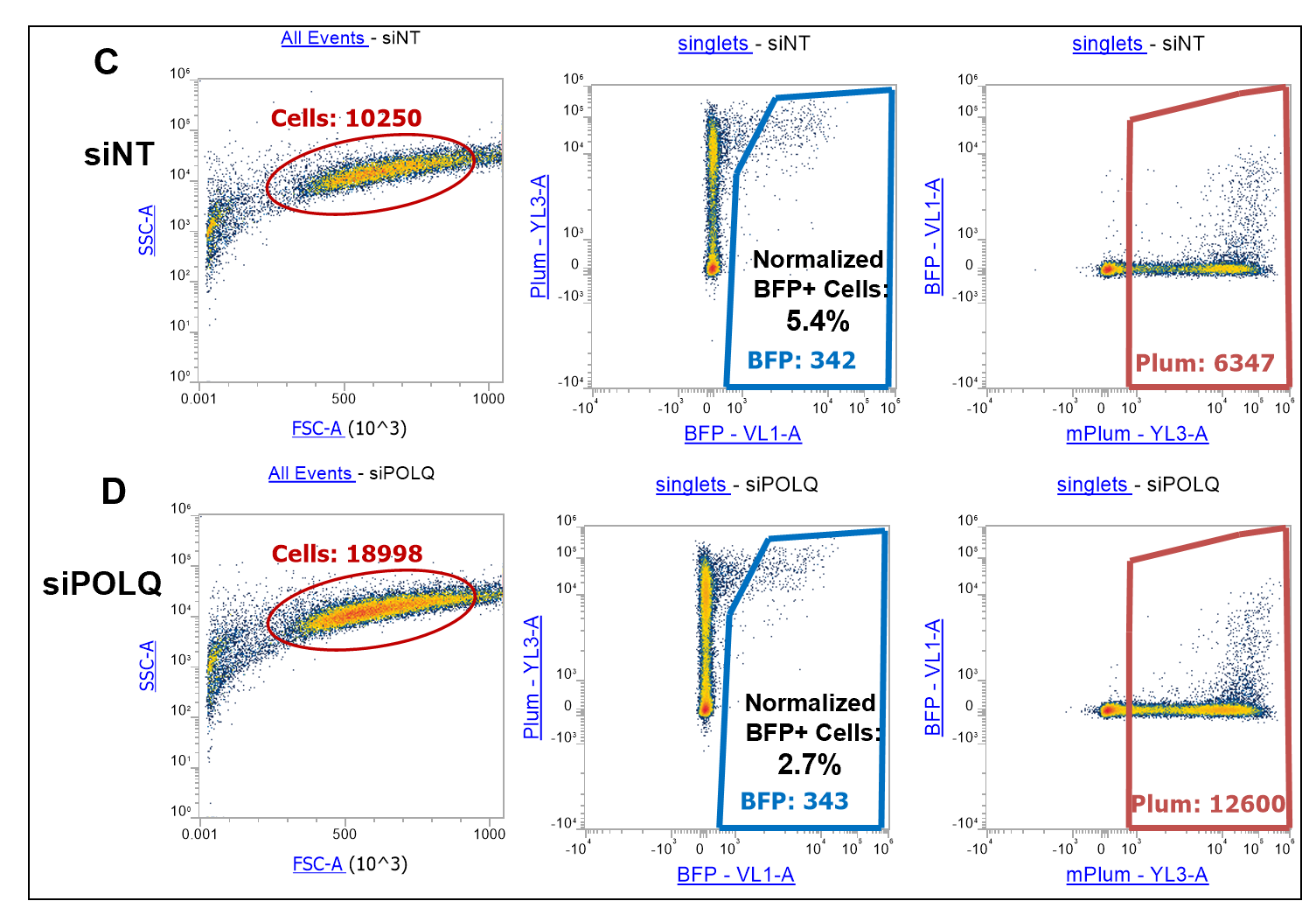

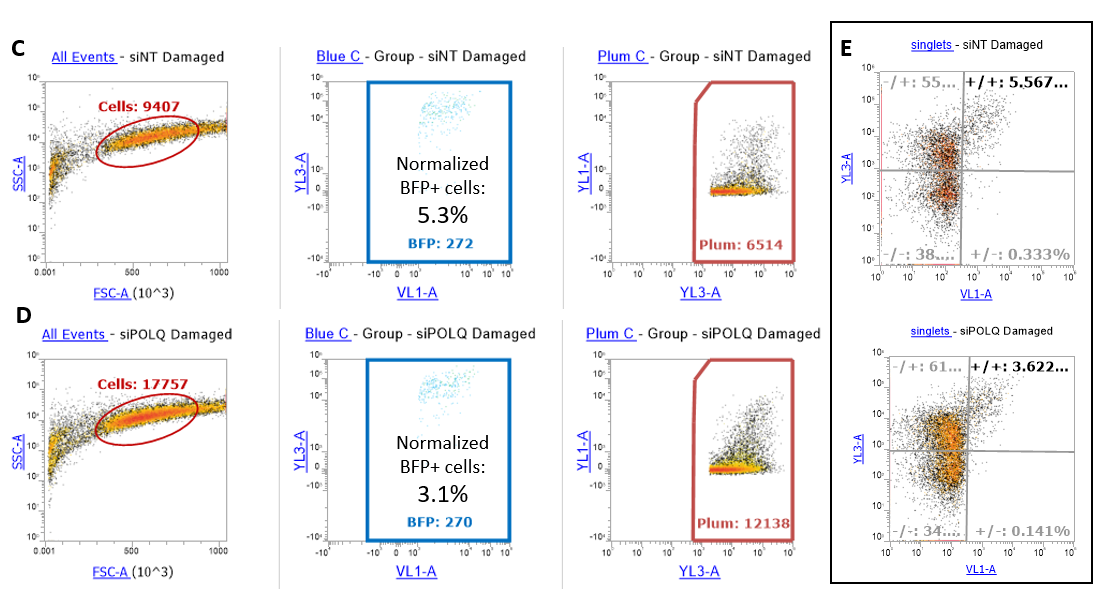
**

**Fig. S1.** Cellular validation of BFP_MMEJ8 reporter assay. A) U251 cells were electroporated with closed circular BFP_MMEJ8 plasmid, mPlum transfection control, and non-fluorescent carrier DNA. B) same as A, except BFP_MMEJ8 was linearized *in vitro* by ScaI prior to transfection. C and D) U2OS cells were transfected with non-targeting siRNA (siNT, panel C) or POLQ-targeting siRNA (siPOLQ, panel D). 72 hr later, cells were electroporated with linearized BFP_MMEJ8 (same amounts as in B). The normalized percentage of BFP+ cells (100 × BFP+ cells/Plum+ cells) is displayed for each condition. E) Same data from C and D, except a different gating strategy is shown, resulting in slightly different values for the percentage of MMEJ events (top right quadrant). We note that “Reporter expression,” the metric we use for FM-HCR assays, integrates both the percentage of fluorescent cells and the mean fluorescence intensity (see Experimental).


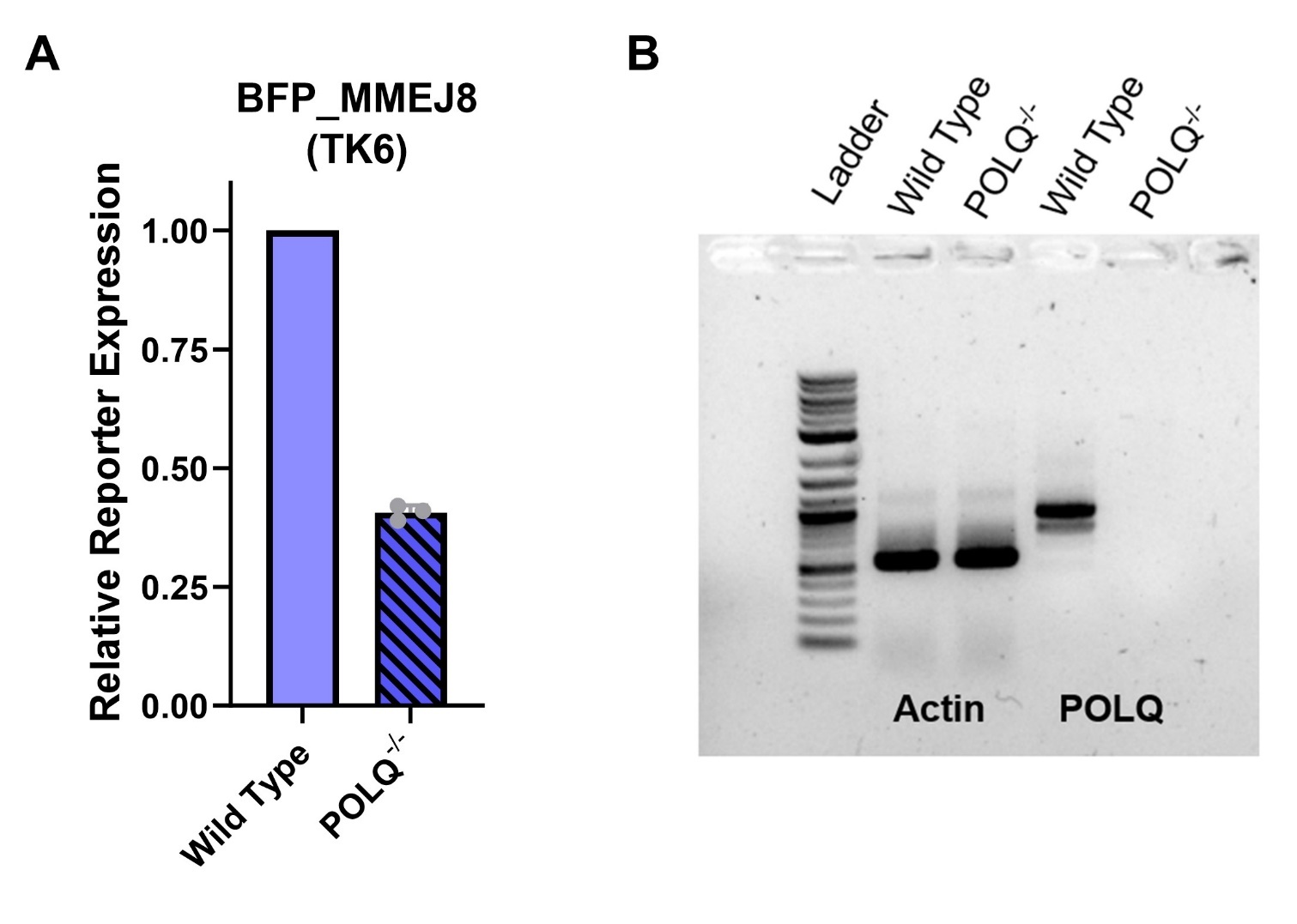


**Figure S2.** Cellular validation of BFP_MMEJ8 reporter in TK6 cells and confirmation of POLQ knockdown by qRT-PCR. A) TK6 cells were electroporated with BFP_MMEJ8 reporter and assessed by flow cytometry 24 hr later. Data are presented as the mean of three independent experiments and error bars show the std. dev. B) Confirmation of POLQ knockout in TK6 cells by agarose gel analysis of RT-PCR products. POLQ knockout cells (reported previously by Akagawa et. al.)^1^ were generated by CRISPR-Cas9 targeting and homology-directed repair to delete exons 20-22 in the polymerase domain. Primers within this region were used to amplify cDNA isolated from wild type or POLQ^-/-^ cells. Primers for amplifying actin were used as input control.


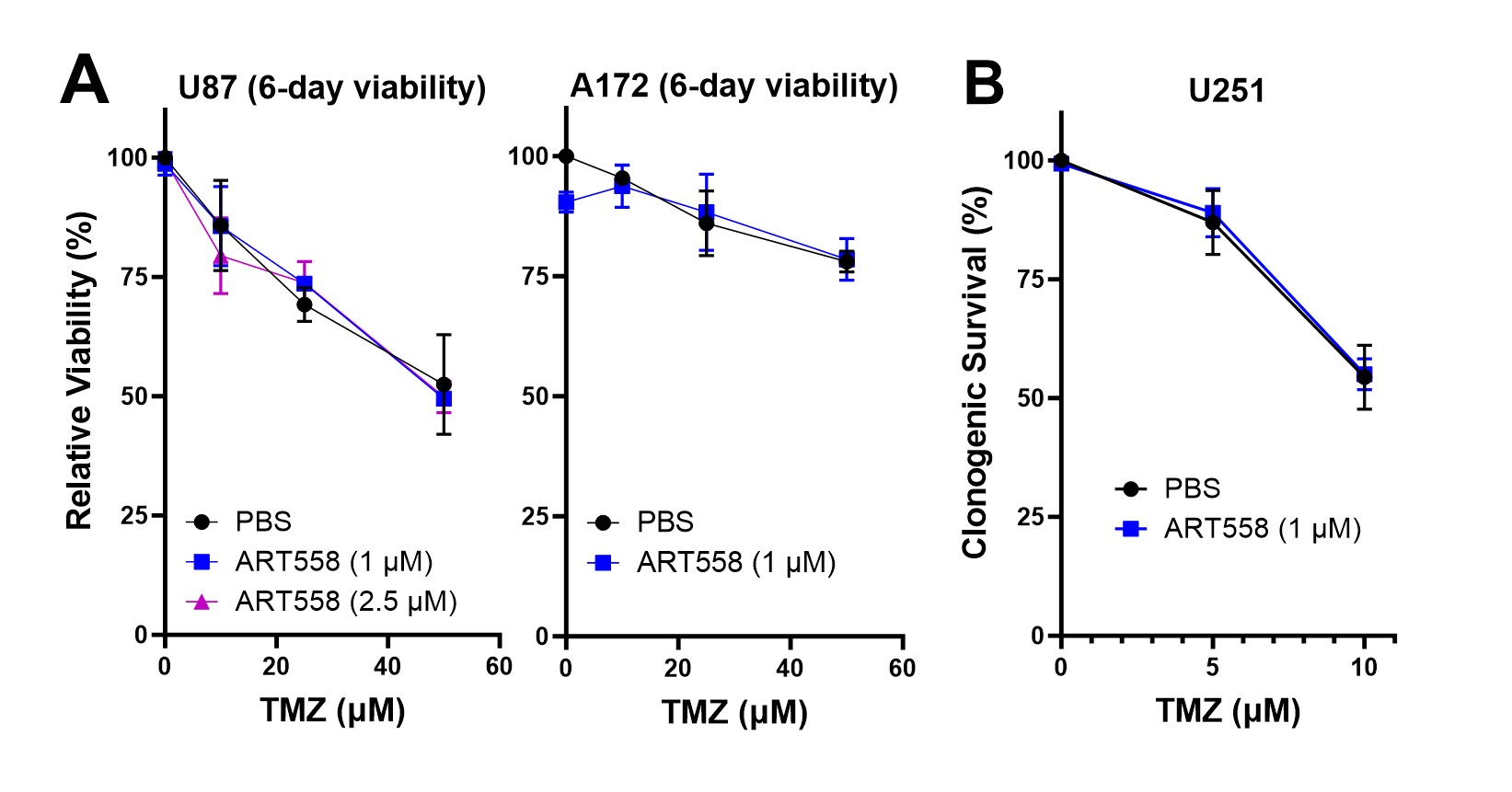


**Fig. S3.** ART558 does not potentiate TMZ in GBM cell lines. A) Relative viability of GBM cell lines treated with the indicated dose of TMZ and co-treated with vehicle (PBS) or ART558 for 6 days. Data are presented as the mean of three independent experiments (which each had three technical replicates) and error bars show the std. dev. B) Clonogenic survival of U251 cells treated with PBS or ART558 along with the indicated dose of TMZ for 4 days followed by media replacement and colony growth for 10 additional days. Data are the mean of three independent experiments (which each had three technical replicates), and error bars show the standard deviation.

**
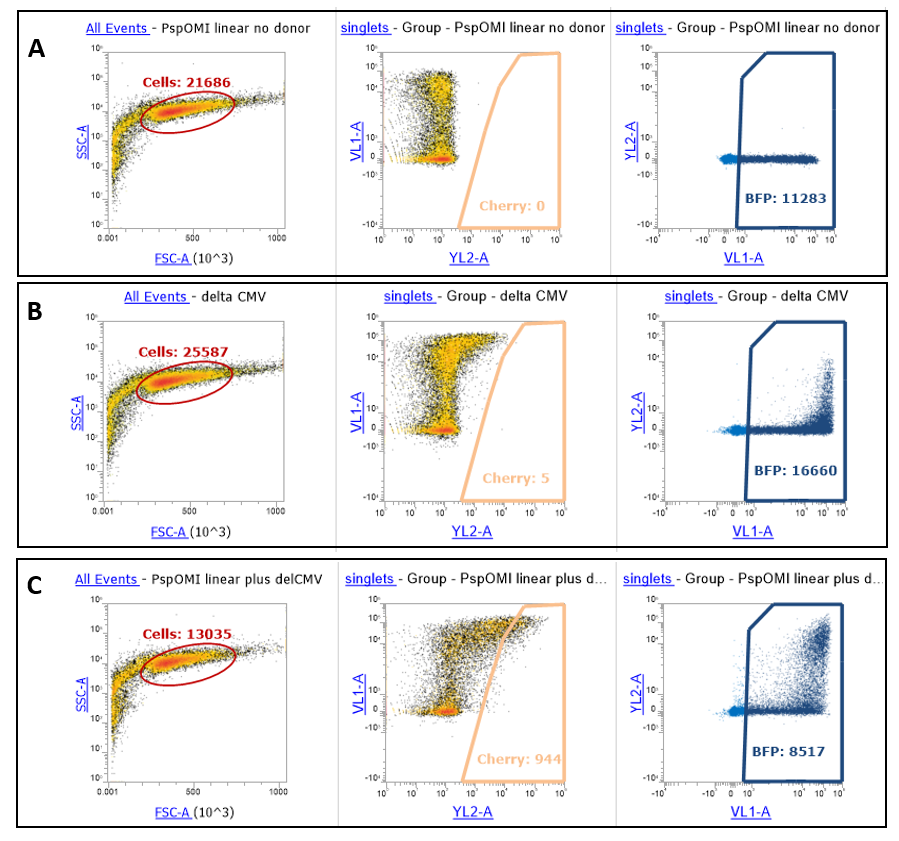
**

**
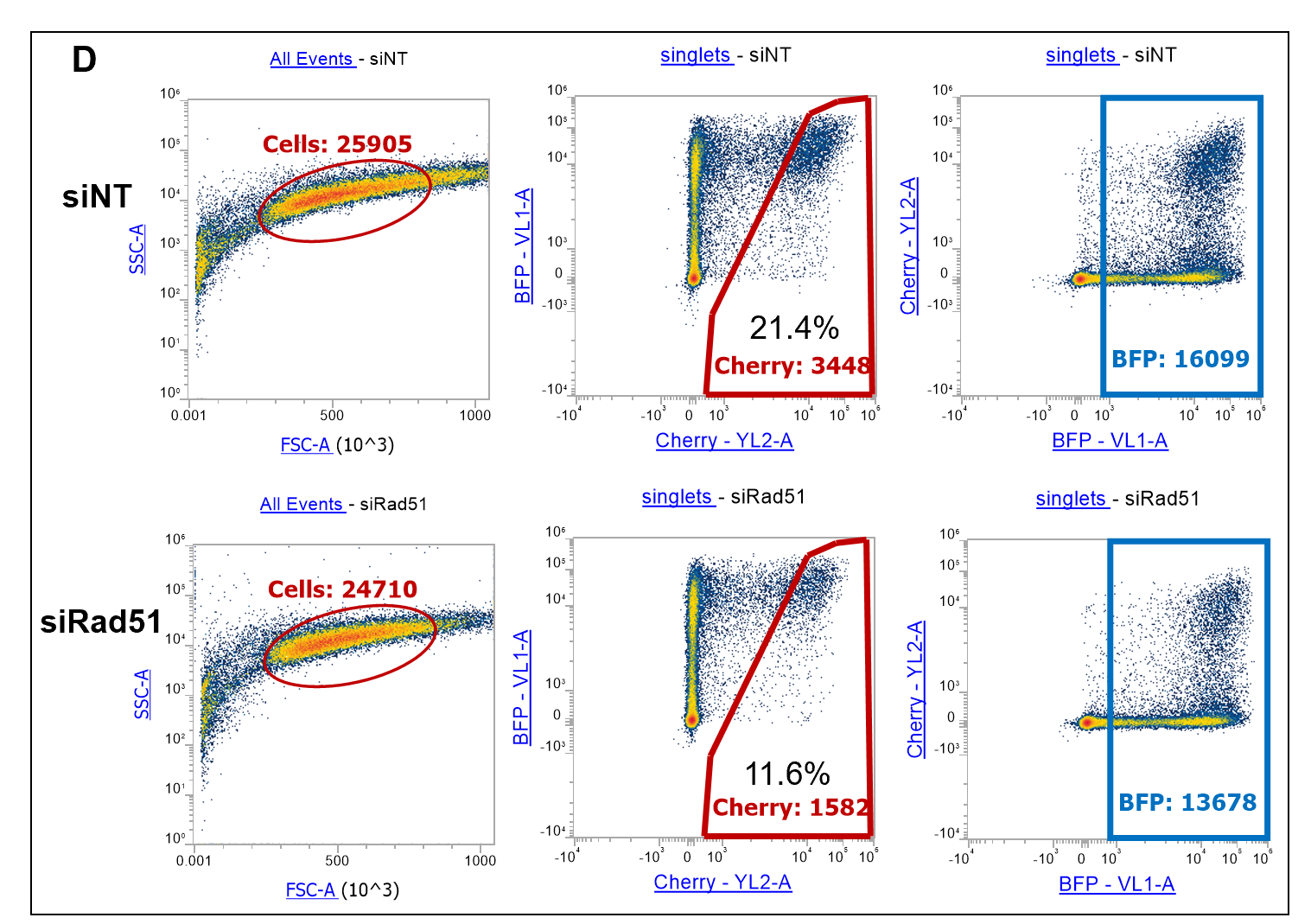
**

**Fig. S4.** Validation of PspOMI Cherry HR reporter in U2OS cells. Transfection with A) linearized PspOMI Cherry plasmid (100 ng), pMax BFP transfection control (100 ng), and non-fluorescent carrier plasmid pcx nnx Δ3 (1000 ng). B) ΔCMV Cherry plasmid (1000 ng) and pMax BFP (100 ng). C) HR cocktail: linearized PspOMI Cherry plasmid (100 ng), ΔCMV Cherry plasmid (1000 ng), and pMax BFP (100 ng). D) PspOMI Cherry HR assay in U2OS cells transfected with non-targeting siRNA (siNT) or Rad51 knockdown (siRad51) followed by transfection with PspOMI Cherry HR cocktail using BFP as a transfection control. Normalized percentage of Cherry HR-positive cells is displayed. Fluorescent channels are as follows VL1: BFP, YL2: mCherry

**
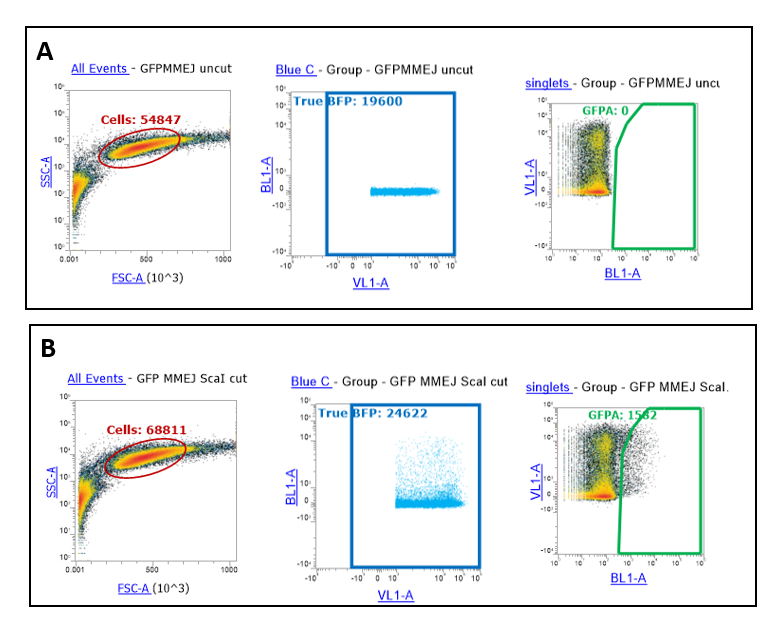
** **
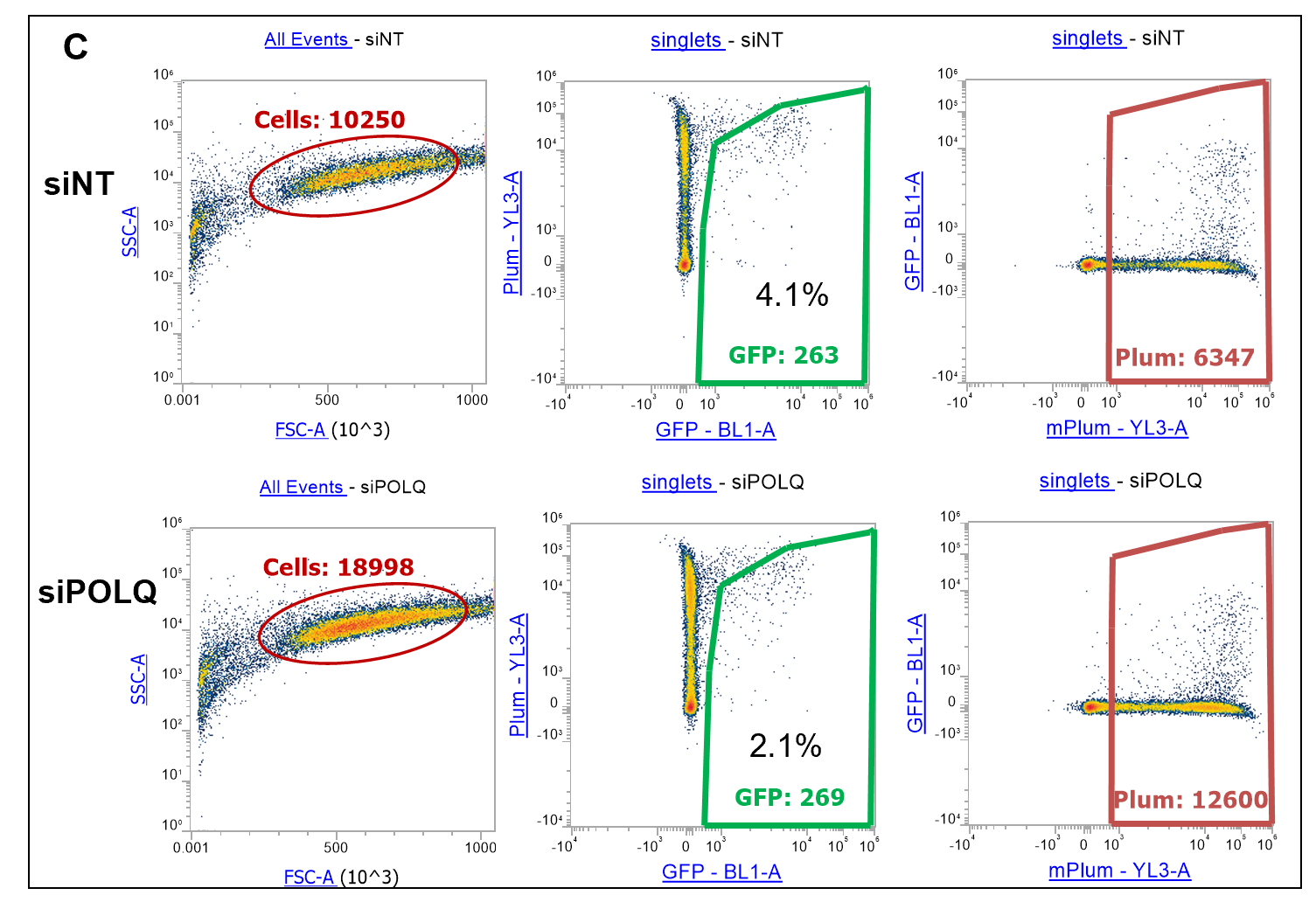
**

**Fig. S5.** Validation of GFP_MMEJ6 reporter in U2OS cells. A) Transfection of U2OS cells with undigested GFP_MMEJ6 (250 ng), carrier plasmid (1000 ng), and pMax BFP (100 ng) transfection control. B) Transfection with ScaI-digested GFP_MMEJ6 (250 ng), carrier plasmid (1000 ng), and pMax BFP transfection control (100 ng). C) Transfection of BFP_MMEJ8 (250 ng), GFP_MMEJ6 (250 ng) and pMax mPlum transfection control (100 ng) plus carrier plasmid (1000 ng) into U2OS cells transfected with non-targeting siRNA (siNT) or POLQ knockdown (siPOLQ) 48 hr prior. Normalized GFP-positive cells are shown for each. Note: this is the same transfection displayed in Fig. S1, except only GFP and Plum gates are shown here. See Fig. S1 for BFP_MMEJ8 plots. Fluorescent channels are as follows VL1: BFP, BL1: GFP, YL3: mPlum


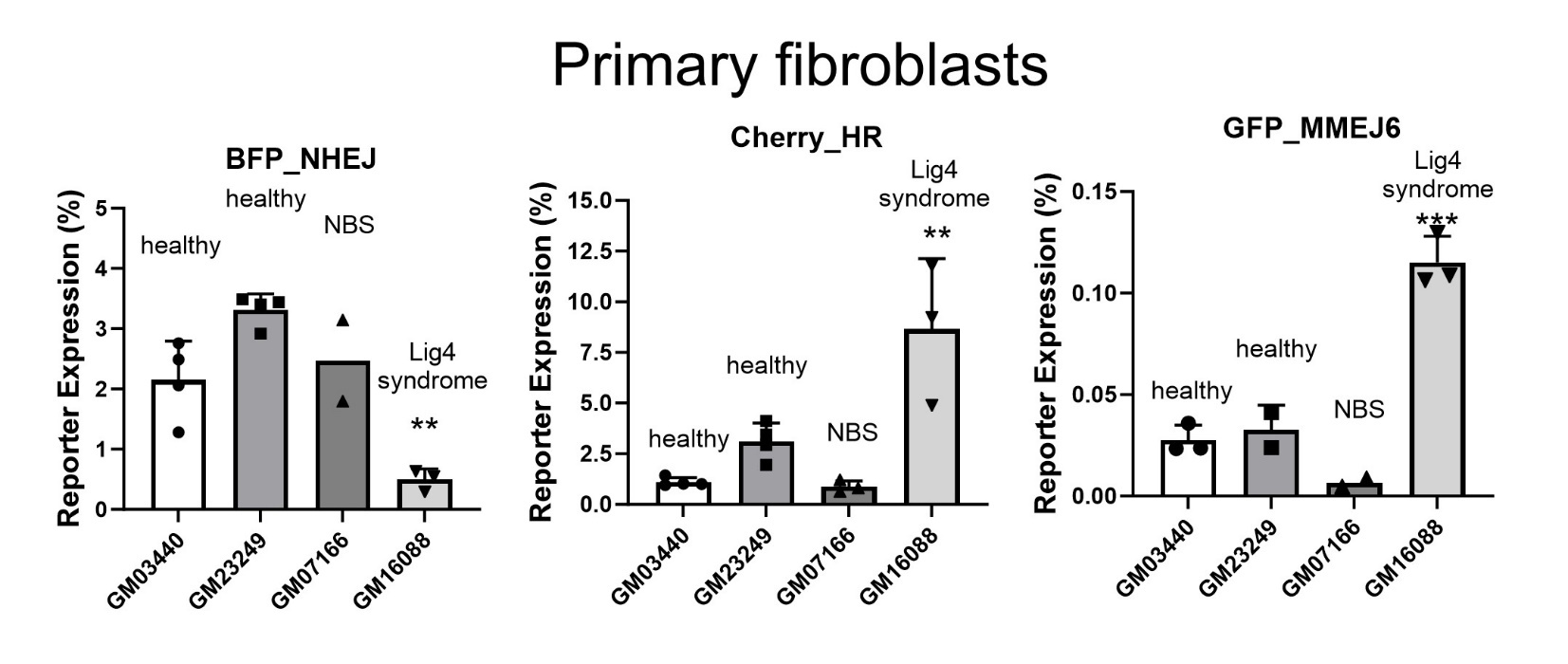


**Fig. S6.** FM-DSBR analysis in primary fibroblasts from the Coriell Institute. Healthy individuals (GM03440 and GM23249) were compared to an individual with Nijmegen breakage syndrome (NBS, GM07166) and an individual with Lig4 syndrome (GM16088). Data are the mean of three independent experiments, except for GM07166 which had only two experiments for NHEJ and MMEJ before cells ceased growth. Error bars show the standard deviation. Asterisks represent p-values from comparison to GM03440 by unpaired two-tailed t-test. **: p<0.01, ***: p<0.001


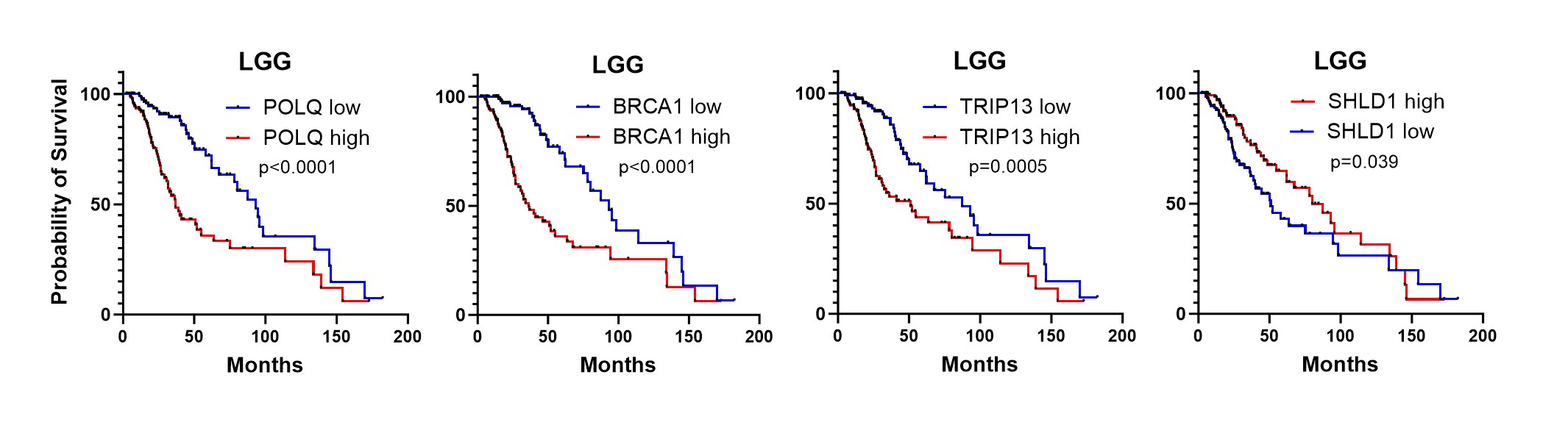


**Fig. S7.** Overall survival of low grade glioma (LGG) patients from The Cancer Genome Atlas dataset receiving TMZ and stratified by median expression of the indicated gene. Data from TCGA LGG dataset were analyzed using cBioPortal. p-values are from Mantel-Cox test.


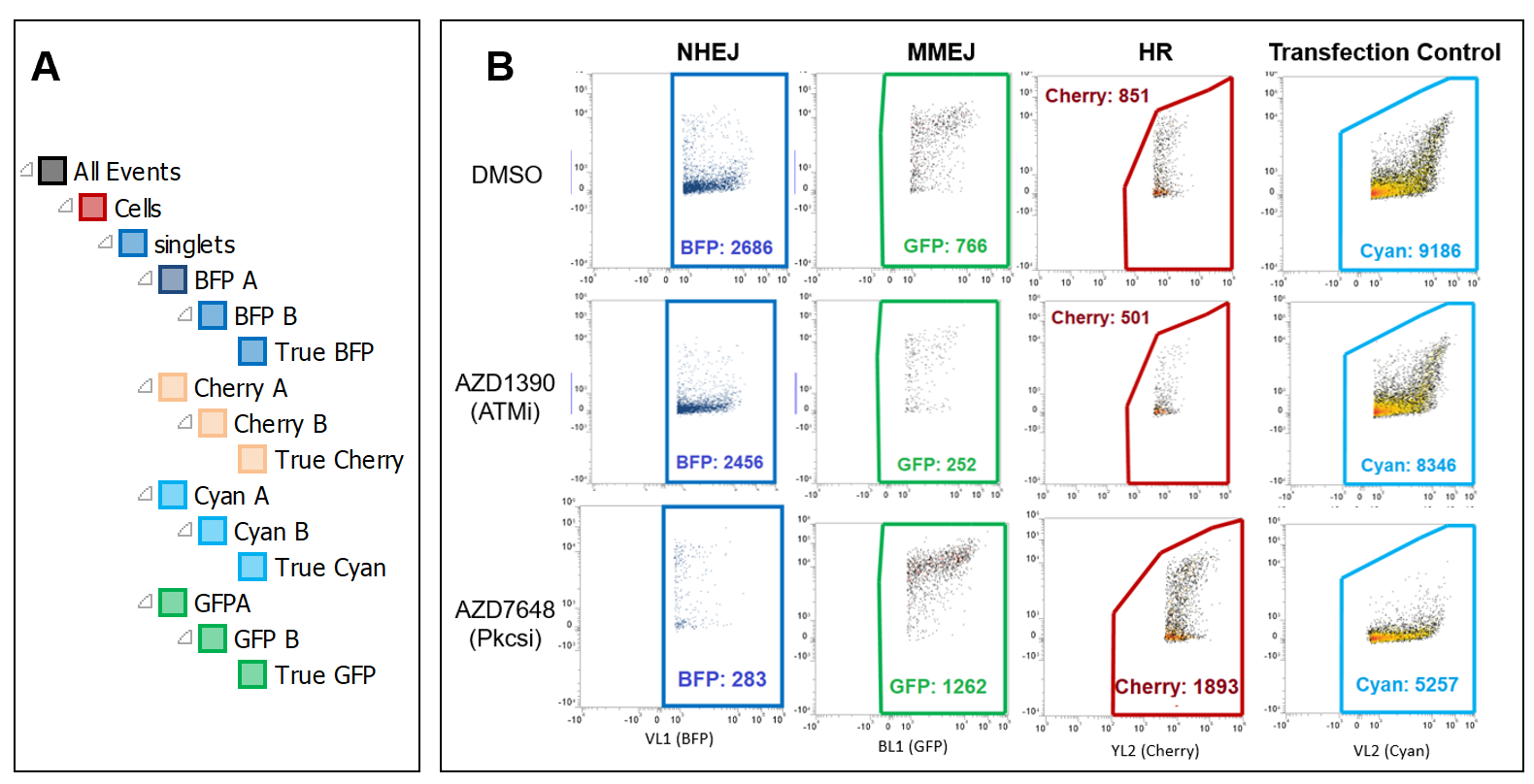


**Fig. S8. Representative flow cytometry plots from FM-DSBR screen in U251 cells.** A) Gating hierarchy used in FM-DSBR experiments. Gating in FM-HCR experiments is described in detail in Piett et. al.^2^ Briefly, events were gated based on forward scatter area and side scatter area to identify the population of cells. Cells were gated by forward scatter pulse width versus forward scatter pulse height to exclude doublets and identify the population of singlet cells. The population of “True” events in each fluorescent channel was determined by plotting each fluorescent channel against every other channel, ex: VL1-BFP vs BL1-GFP (gate BFP A), VL1-BFP vs. YL2-Cherry (gate BFP B) and VL1-BFP vs. VL2-Am Cyan (True BFP). Single color control transfections consisting of one reporter plasmid (BFP, GFP, mCherry, or AmCyan) were conducted and gating and compensation were established such that fluorescence in each channel was independent of any other channel. B) Representative flow cytometry plots for FM-DSBR in U251. U251 cells were pre-treated with DMSO, AZD1390, or AZD7648 for 2 hr and transfected with BFP_NHEJ, GFP_MMEJ6, Cherry_HR, and AmCyan (transfection control) and analyzed by flow cytometry after 24 hr.


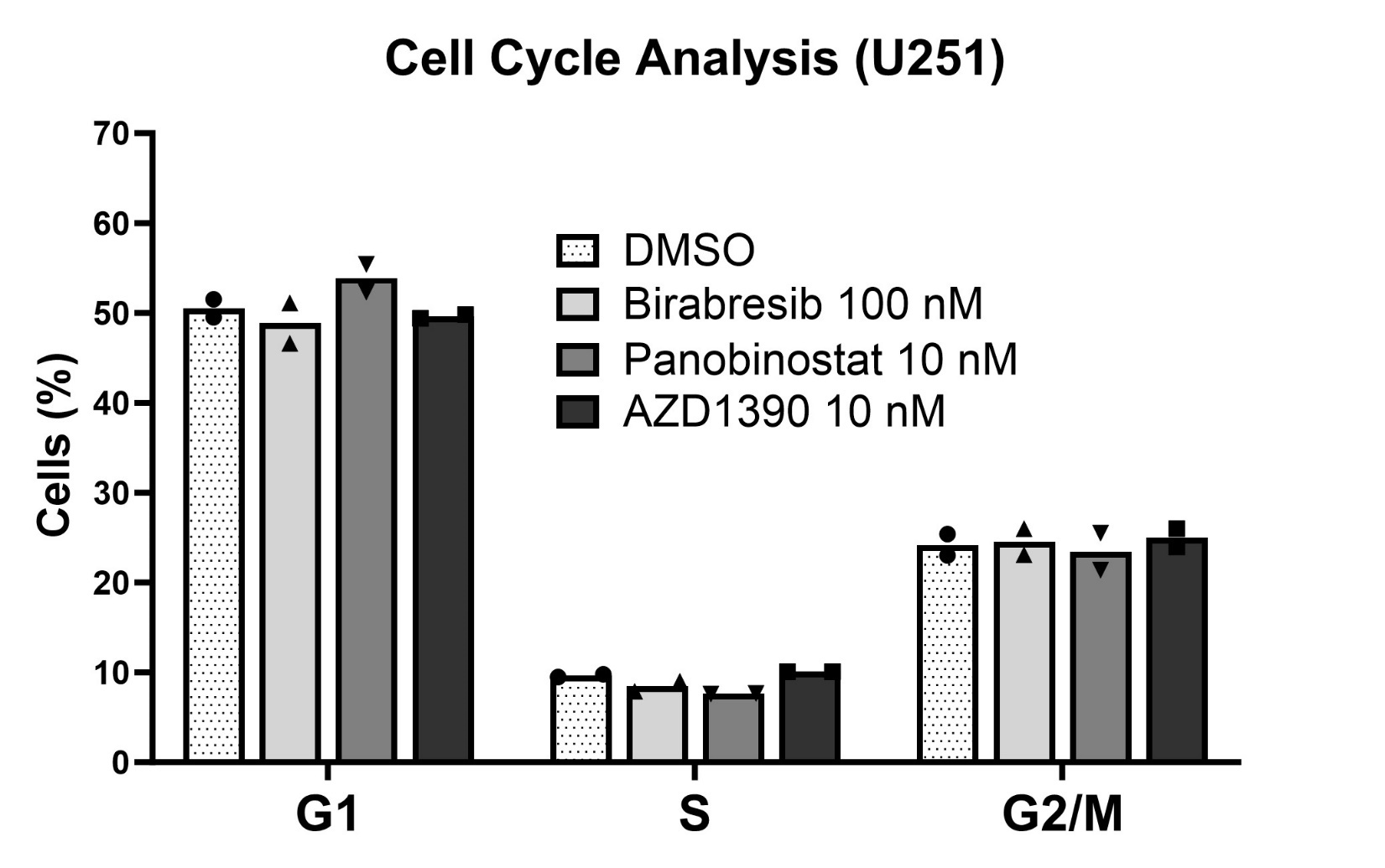


**Figure S9.** AZD1390, Birabresib, and Panobinostat do not perturb the cell cycle of U251 cells under conditions used in FM-DSBR screen**.** U251 cells were treated with the indicated agent for 24 hr, collected, fixed and stained with propidium iodide and analyzed by flow cytometry as described in methods. Each data point is from an independent experiment (n=2).


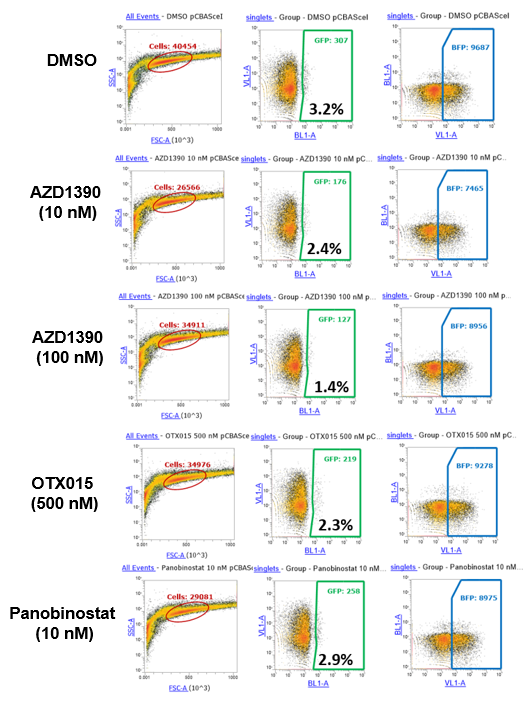


**Fig. S10.** Representative flow cytometry plots for U251 DR-GFP reporter cells. Cells were pre-treated for 2 hr with inhibitor and transected with pCBASceI plasmid (500 ng) and BFP transfection control (10 ng) and analyzed by flow cytometry 72 hr later. The normalized percentage of GFP-positive cells is displayed for each condition. Fluorescent channels are as follows VL1: BFP, BL1: GFP.


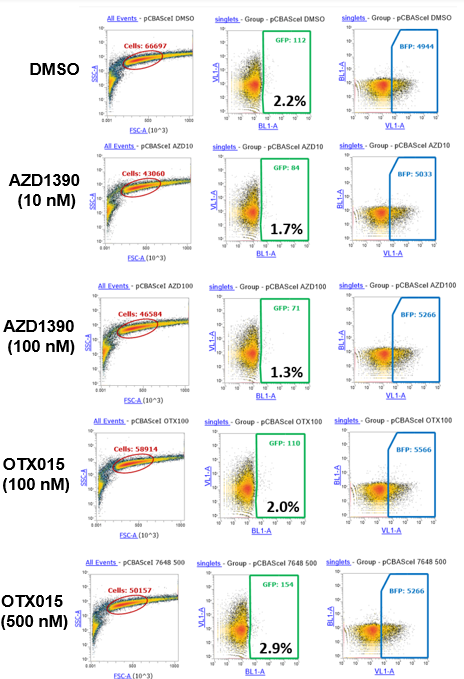


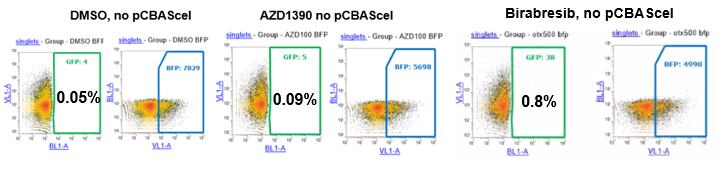


**Fig. S11.** Representative flow cytometry plots for U251 EJ2-GFP reporter cells. Cells were pre-treated for 2 hr with inhibitor and transected with pCBASceI plasmid (500 ng) and BFP transfection control (10 ng) and analyzed by flow cytometry 72 hr later. The normalized percentage of GFP-positive cells is displayed for each condition. Controls transfected with pmax BFP but not pCBASceI plasmid are shown at the bottom for DMSO, AZD1390, and Birabresib. Fluorescent channels are as follows VL1: BFP, BL1: GFP,


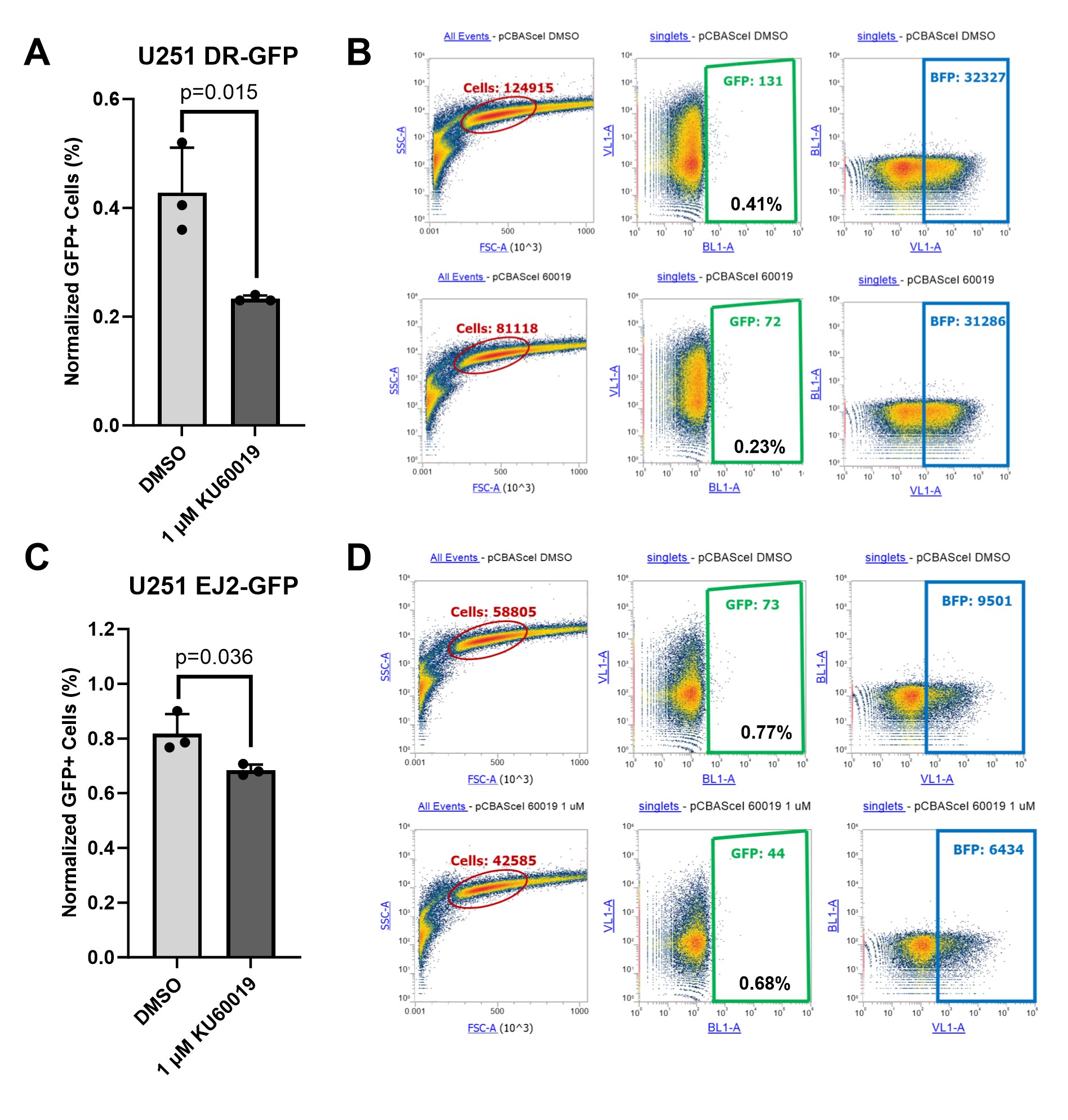


**Figure S12.** The ATM inhibitor KU60019 inhibits HR and MMEJ in U251. A) Analysis of HR in the U251 DR-GFP reporter cell line. Each data point represents an independent experiment. B) Representative flow cytometry plots from A. C) Analysis of MMEJ in the U251 EJ2-GFP reporter cell line. Each data point represents an independent experiment. D) Representative flow cytometry plots from C. In both reporter cell lines, cells were treated with DMSO or KU60019 and immediately transfected with pCBASceI plasmid (1 µg) and pMax BFP plasmid (10 ng). Cells positive for GFP were counted as HR or MMEJ events respectively. and the number of GFP positive cells was divided by the number of BFP positive cells and multiplied by 100 to calculate “Normalized GFP-positive cells (%),” displayed within the GFP gate on the representative flow plots. Data are presented as the mean of three independent experiments and p-values are from statistical comparison by unpaired two-tailed t-test. Fluorescent channels are as follows: GFP: BL1, BFP: VL1


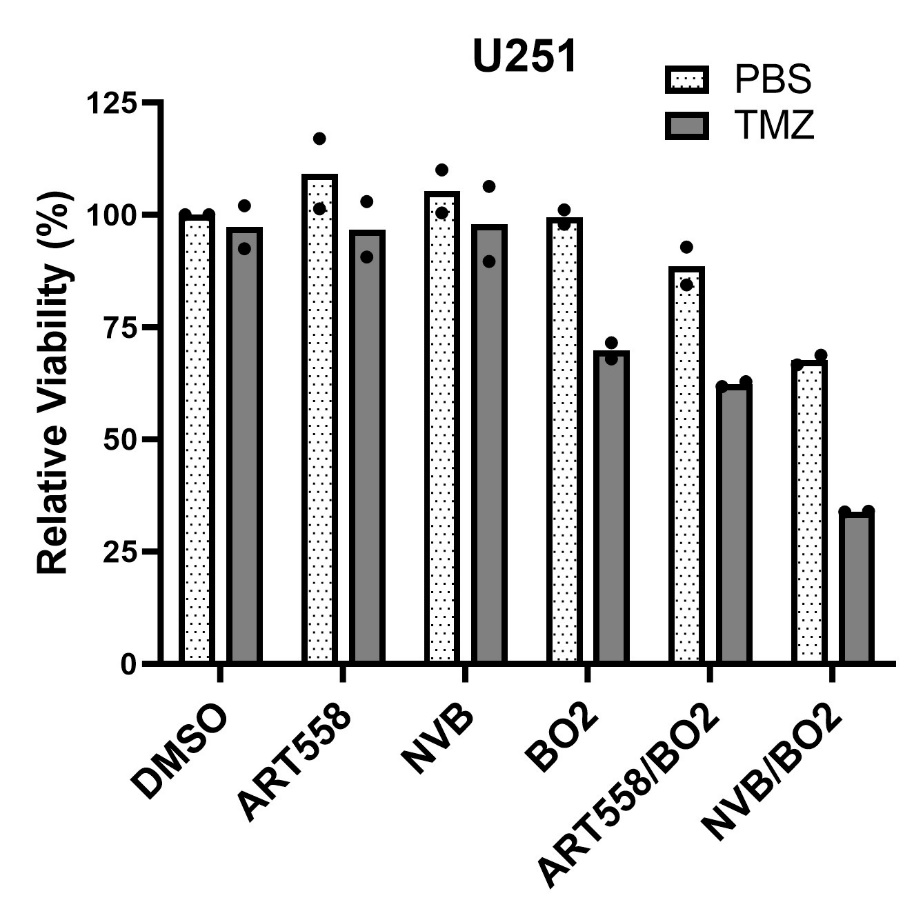


**Figure S13.** Relative viability of U251 cells after 6-day treatment with TMZ (10 µM) and the indicated agent: ART558: 2.5 µM, NVB, 50 µM, BO2, 10 µM. The mean of two independent experiments (conducted in triplicate) is displayed, and each data point is from an independent experiment.


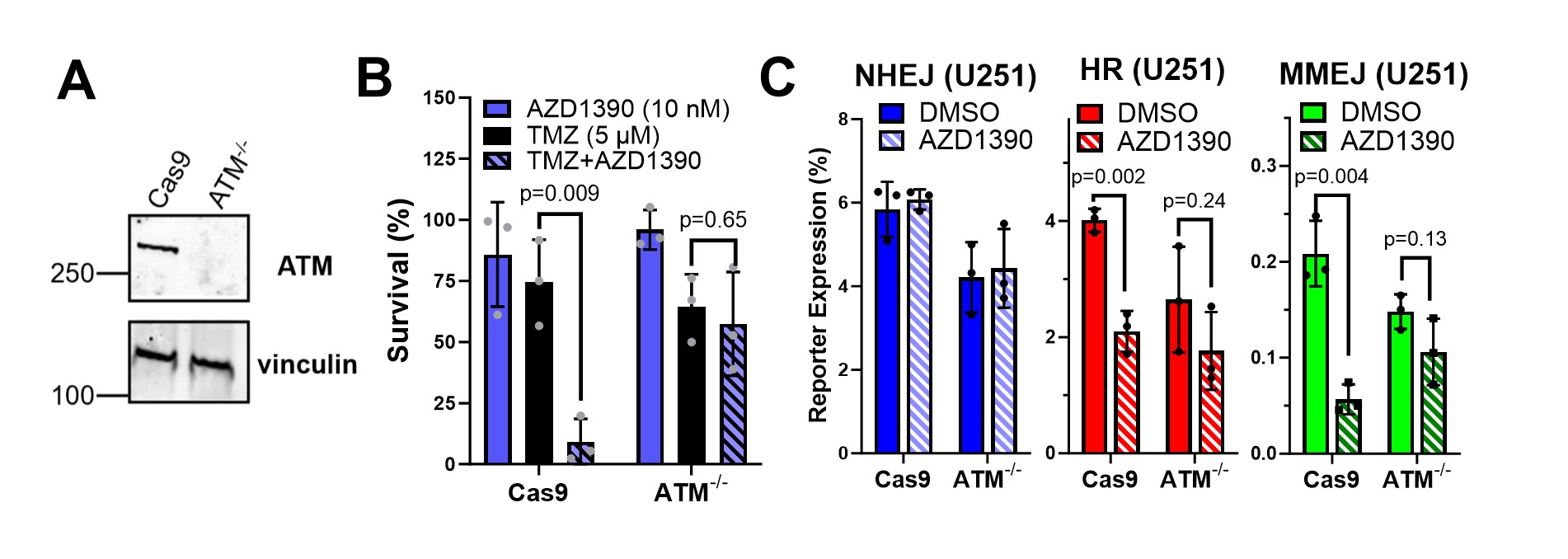


**Figure S14.** ATM knockout markedly ablates the treatment-enhancing effects of AZD1390. A) Western blot of U251-Cas9 cells and ATM^-/-^ cells. B) Clonogenic survival of U251-Cas9 or ATM^-/-^ cells treated with AZD1390, TMZ, or TMZ+AZD1390 for 4 days followed by media replacement and colony growth until visible by eye. C) FM-DSBR in U251-Cas9 or U251 ATM^-/-^ cells treated with AZD1390 (100 nM). In B and C, data are presented as the mean of three independent experiments, and error bars show the std. dev. p-values are from unpaired two-tailed t-test with Holm-Sidak correction for multiple testing.


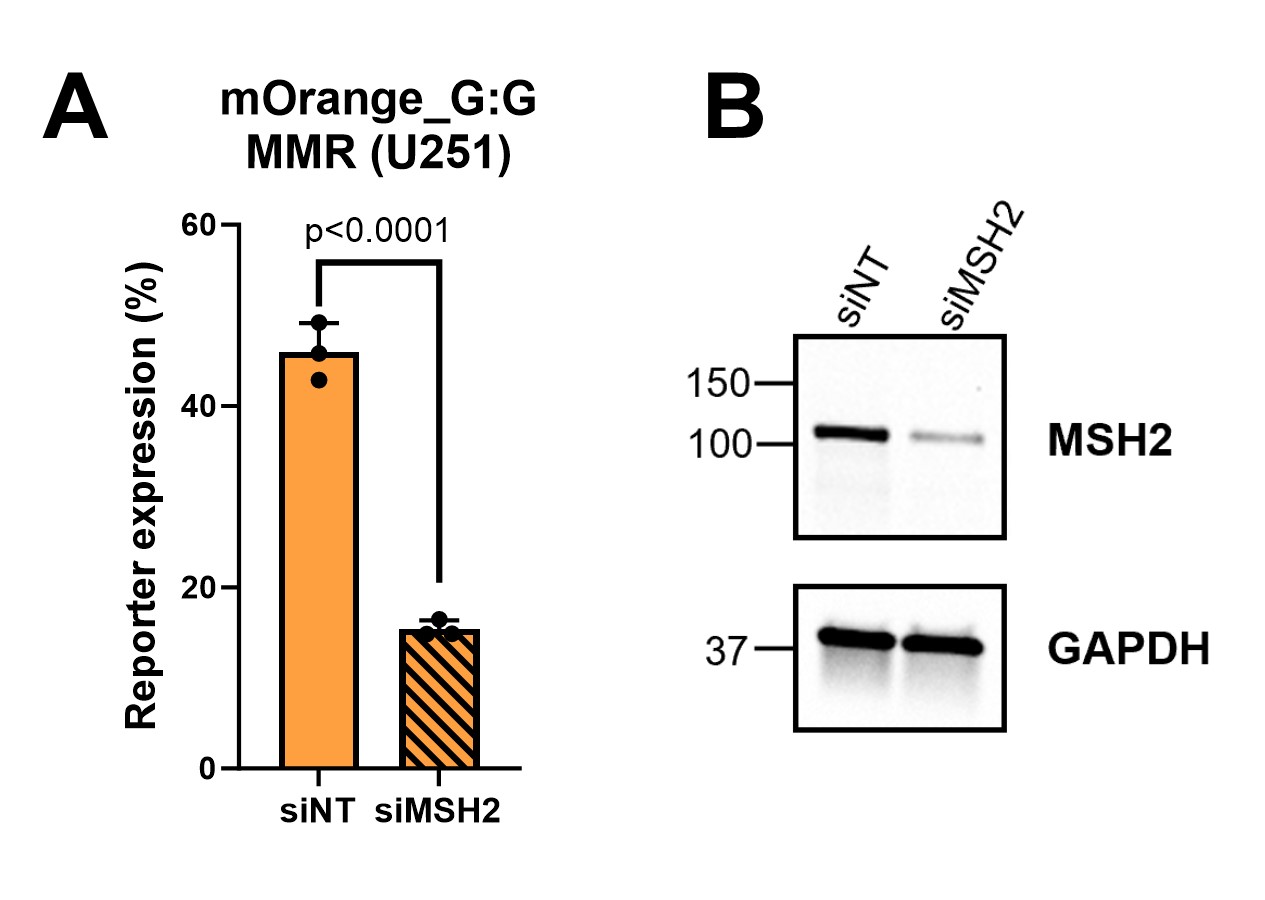


**Figure S15.** Knockdown of MSH2 reduces MMR capacity of U251 cells. A) G:G MMR capacity using mOrange_G:G. The mean of three independent experiments is plotted, error bars show the standard deviation, and p-value is from unpaired two-tailed t-test. B) Western blot of U251 cells transfected with 10 pmol of non-targeting siRNA (siNT) or MSH2-targeting siRNA and collected 72 hr later.


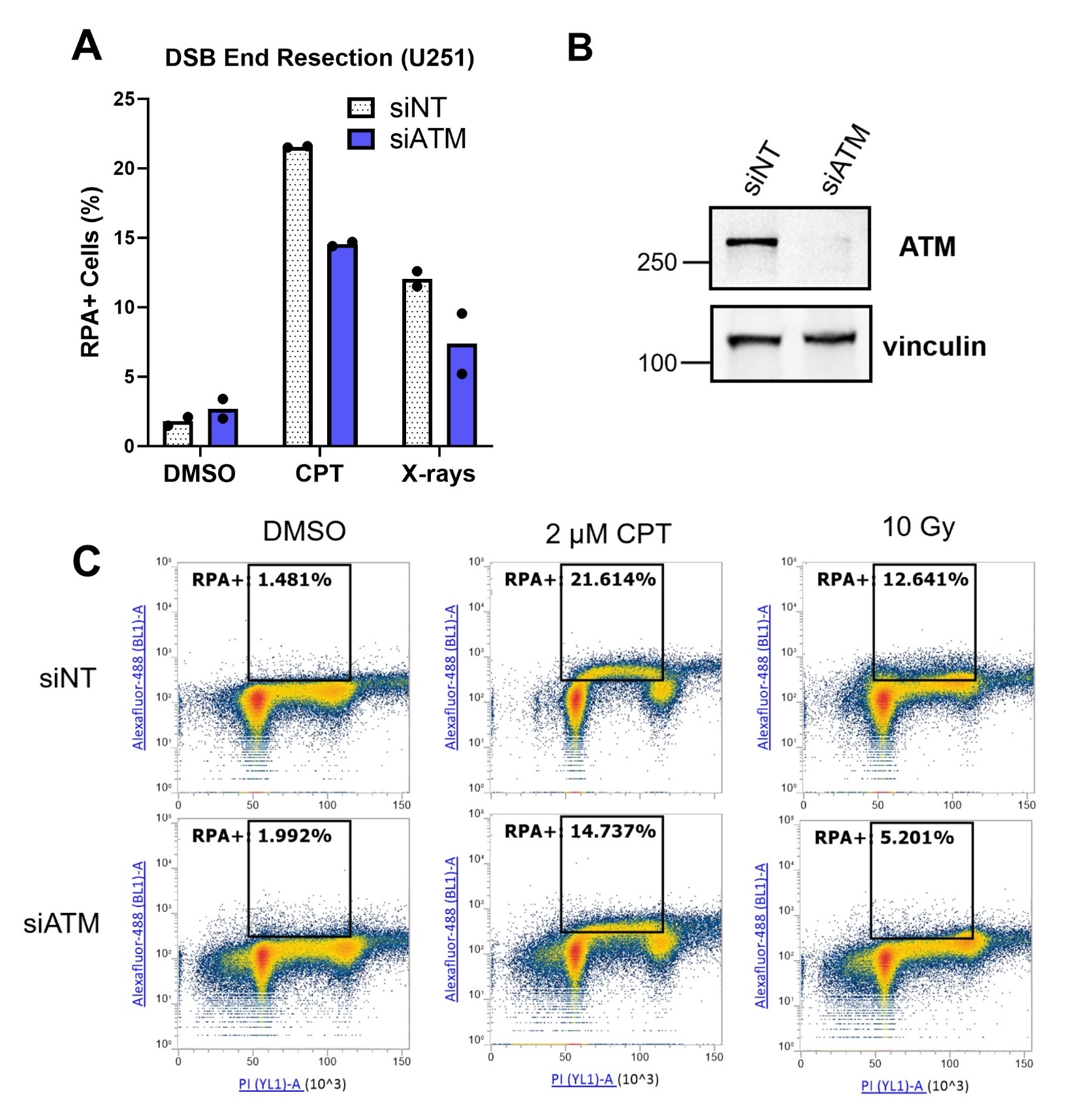


**Figure S16.** ATM knockdown suppresses DNA damage-induced RPA signal in U251 cells. A) DSB end resection as measured by RPA staining. U251 cells were transfected with the indicated siRNA (10 pmol) using Lipofectamine RNAiMax and transferred to 6-well plates 48 hr later. After an additional 48 hr, cells were treated with camptothecin (CPT) or radiation (10 Gy) and collected for RPA immunostaining after 1 hr. Each data point is from an independent experiment (n=2) and then mean is displayed. B) Western blot showing ATM knockdown in A. C) representative flow cytometry plots from A.


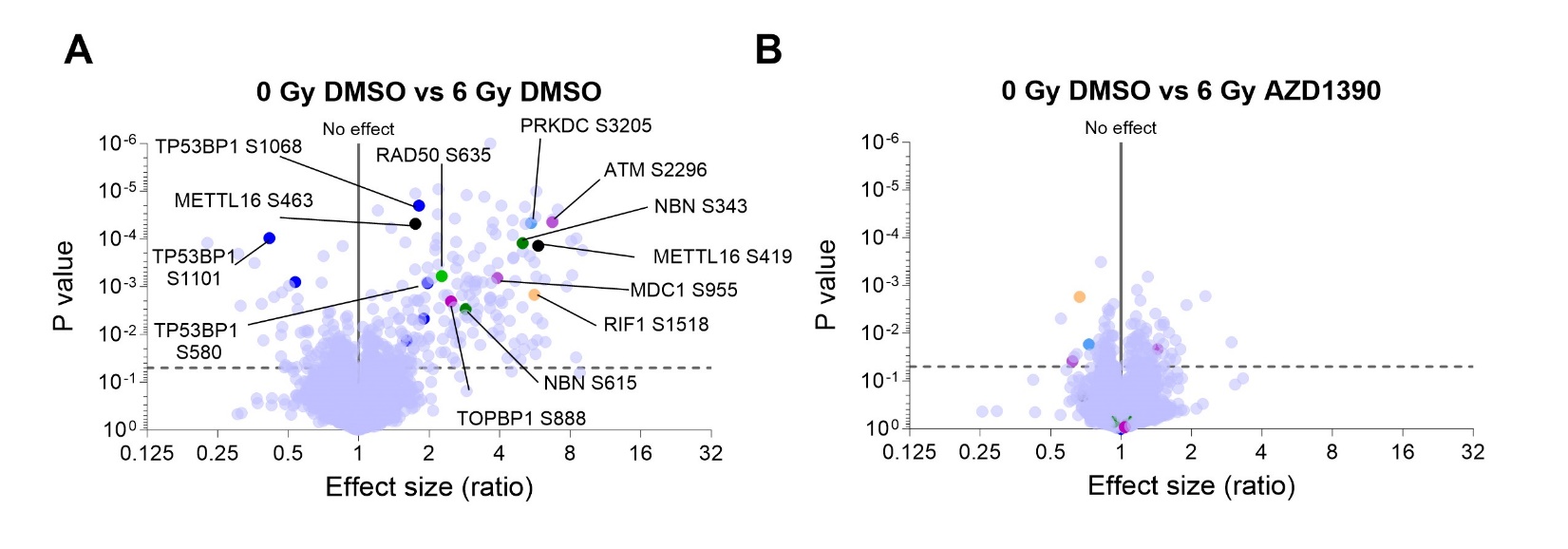


**Figure S17.** A) Volcano plot comparing phospho sites in 0 Gy and 6 Gy condition. B) Volcano plot for 0 Gy and 6 Gy AZD1390. Data are from three biologically independent experiments.


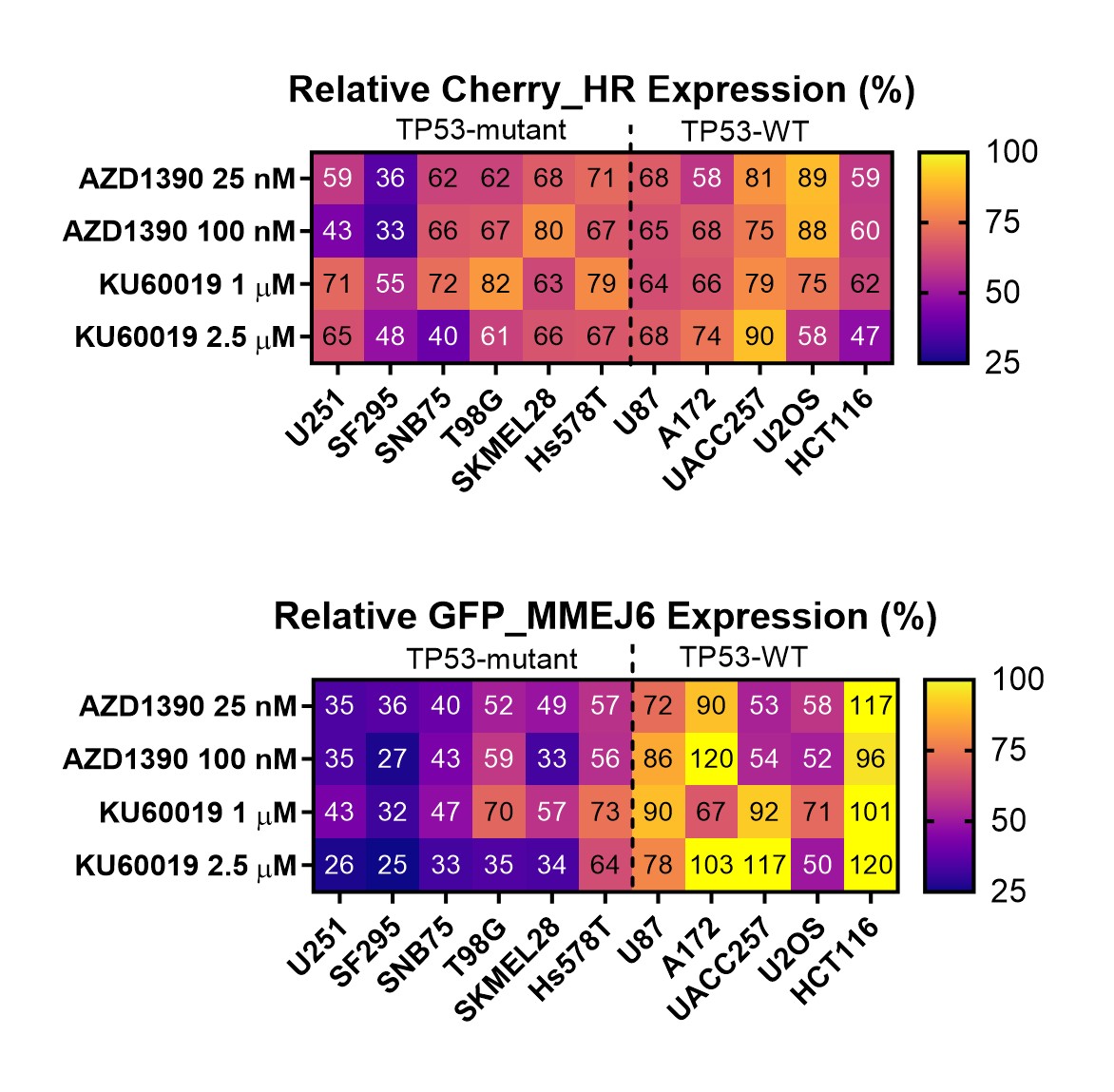


**Fig. S18.** HR and MMEJ analysis in cancer cell lines following AZD1390 or KU60019 treatment at the indicated dose. Data are presented as the mean of four independent experiments normalized to DMSO vehicle.


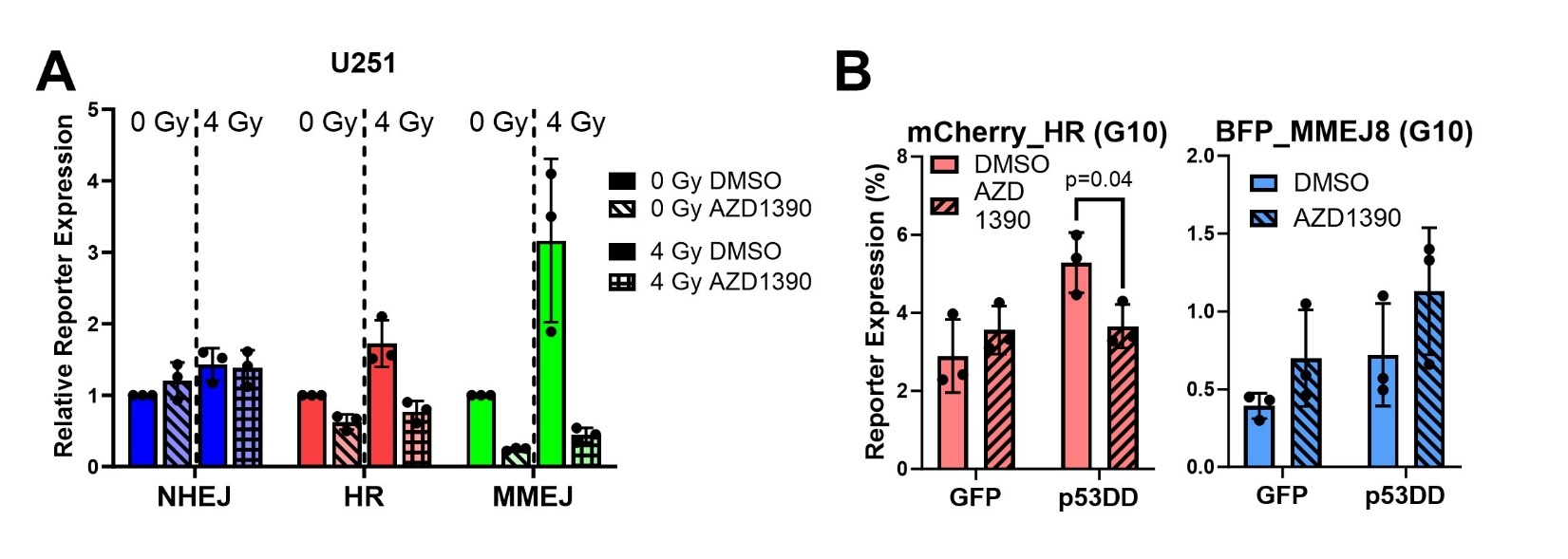


**Fig. S19.** A) FM-DSBR in U251 cells pre-treated with DMSO or AZD1390 (100 nM) for 1 hr, treated with 0 Gy or 4 Gy, and transfected with FM-DSBR reporters 1 hr later. B) mCherry_HR and BFP_MMEJ reporters in G10-GFP and G10-p53DD cells after 1 hr pre-treatment with AZD1390 (100 nM). Data are from three independent experiments, error bars show the standard deviation. In B, p-value is from unpaired two-tailed t-test.

**MMEJ reporter gene sequences**

GFP_MMEJ6 gene sequence 5’ – 3’ (ATG start codon, TGA stop codon). Microhomologies are underlined (5’-TGACCT-3’). ScaI restriction site is bold.

ATGGTGAGCAAGGGCGAGGAGCTGTTCACCGGGGTGGTGCCCATCCTGGTCGAGCTGGACGGCGACGTAAACGGCCACAAGTTCAGCGTGTCCGGCGAGGGCGAGGGCGATGCCACCTACGGCAAGCTGACCCTGAAGTTCATCTGCACCACCGGCAAGCTGCCCGTGCCCTGGCCCACCCTCGTGACCACCCTGACCTAGGCTCG**AGTACT**AGCCCGCTGACCTACGGCGTGCAGTGCTTCAGCCGCTACCCCGACCACATGAAGCAGCACGACTTCTTCAAGTCCGCCATGCCCGAAGGCTACGTCCAGGAGCGCACCATCTTCTTCAAGGACGACGGCAACTACAAGACCCGCGCCGAGGTGAAGTTCGAGGGCGACACCCTGGTGAACCGCATCGAGCTGAAGGGCATCGACTTCAAGGAGGACGGCAACATCCTGGGGCACAAGCTGGAGTACAACTACAACAGCCACAACGTCTATATCATGGCCGACAAGCAGAAGAACGGCATCAAGGTGAACTTCAAGATCCGCCACAACATCGAGGACGGCAGCGTGCAGCTCGCCGACCACTACCAGCAGAACACCCCCATCGGCGACGGCCCCGTGCTGCTGCCCGACAACCACTACCTGAGCACCCAGTCCGCCCTGAGCAAAGACCCCAACGAGAAGCGCGATCACATGGTCCTGCTGGAGTTCGTGACCGCCGCCGGGATCACTCTCGGCATGGACGAGCTGTACAAGTGA

BFP_MMEJ8 5’ – 3’ (ATG start codon, TGA stop codon). Microhomologies are underlined ( (5’-TTCCTCTA-3’). ScaI restriction site is bold.

ATGAGCGAGCTGATTAAGGAGAACATGCACATGAAGCTGTACATGGAGGGCACCGTGGACAACCATCACTTCAAGTGCACATCCGAGGGCGAAGGCAAGCCCTACGAGGGCACCCAGACCATGAGAATCAAGGTGGTCGAGGGCGGCCCTCTCCCCTTCGCCTTCGACATCCTGGCTACTAGCTTCCTCT**AGTACT**GTTCCTCTACGGCAGCAAGACCTTCATCAACCACACCCAGGGCATCCCCGACTTCTTCAAGCAGTCCTTCCCTGAGGGCTTCACATGGGAGAGAGTCACCACATACGAAGACGGGGGCGTGCTGACCGCTACCCAGGACACCAGCCTCCAGGACGGCTGCCTCATCTACAACGTCAAGATCAGAGGGGTGAACTTCACATCCAACGGCCCTGTGATGCAGAAGAAAACACTCGGCTGGGAGGCCTTCACCGAGACGCTGTACCCCGCTGACGGCGGCCTGGAAGGCAGAAACGACATGGCCCTGAAGCTCGTGGGCGGGAGCCATCTGATCGCAAACATCAAGACCACATATAGATCCAAGAAACCCGCTAAGAACCTCAAGATGCCTGGCGTCTACTATGTGGACTACAGACTGGAAAGAATCAAGGAGGCCAACAACGAGACCTACGTCGAGCAGCACGAGGTGGCAGTGGCCAGATACTGCGACCTCCCTAGCAAACTGGGGCACAAGCTTAATTGA

**Fig. S20.** Sequences of MMEJ reporters.

**
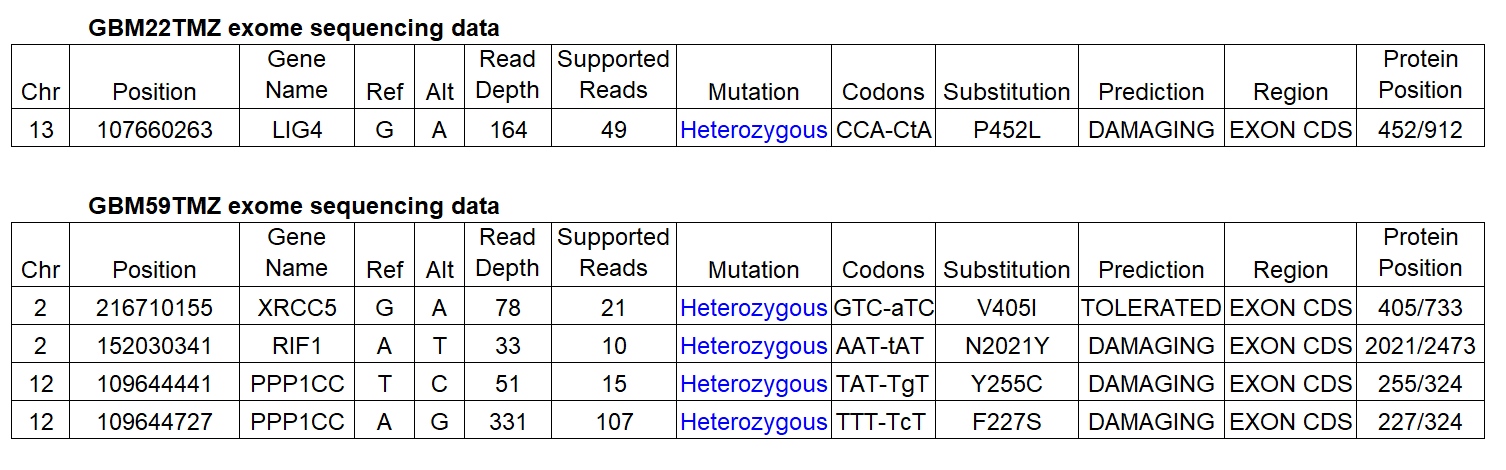
**

**Table S1.** Selected exome sequencing results from GBM22-TMZ and GBM59-TMZ.

| **Gene** | Median Survival Low Expression group | Median Survival High expression group | p-value | q-value |
| --- | --- | --- | --- | --- |
| BRCA1 | **16.64 (14.04 - 22.19)** | **14.96 (12.99 - 18.02)** | 0.09 | 0.14 |
| BRCA2 | **16.60 (13.35 - 20.32)** | **15.12 (14.53 - 19.82)** | 0.92 | 0.92 |
| MRE11 | **14.93 (14.04 - 19.66)** | **15.95 (13.78 - 19.82)** | 0.93 | 0.93 |
| RBBP8 | **15.39 (13.78 - 25.18)** | **15.95 (14.53 - 18.08)** | 0.39 | 0.39 |
| RAD50 | **15.95 (14.04 - 22.19)** | **15.78 (14.93 - 18.08)** | 0.57 | 0.57 |
| NBN | **14.04 (12.66 - 19.66)** | **16.64 (15.39 - 25.18)** | 0.06 | 0.08 |
| RAD51 | **15.95 (13.35 - 20.78)** | **15.12 (14.53 - 19.66)** | 0.92 | 0.92 |
| RAD51B | **15.12 (13.78 - 21.30)** | **16.60 (14.20 - 19.23)** | 0.83 | 0.83 |
| RAD51C | **15.95 (13.35 - 19.82)** | **15.78 (14.20 - 21.30)** | 0.64 | 0.87 |
| RAD51D | **17.88 (15.12 - 22.19)** | **14.73 (12.95 - 17.85)** | 0.25 | 0.37 |
| RAD54L | **16.64 (13.35 - 21.30)** | **14.96 (14.20 - 19.23)** | 0.37 | 0.37 |
| BARD1 | **14.20 (13.12 - 18.08)** | **16.77 (14.96 - 33.67)** | 0.094 | 0.148 |
| PALB2 | **14.93 (13.12 - 20.32)** | **15.95 (14.96 - 19.82)** | 0.43 | 0.92 |
| BRIP1 | **15.95 (13.35 - 20.32)** | **15.78 (14.73 - 19.82)** | 0.87 | 0.87 |
| APEX2 | **16.60 (14.73 - 21.30)** | **15.39 (14.04 - 18.08)** | 0.97 | 0.97 |
| PARP1 | **15.95 (12.95 - 21.30)** | **15.78 (14.53 - 19.23)** | 0.56 | 0.64 |
| FEN1 | **16.64 (13.78 - 25.18)** | **14.93 (14.04 - 18.02)** | 0.06 | 0.08 |
| LIG1 | **16.60 (13.12 - 20.78)** | **15.12 (14.04 - 19.66)** | 0.41 | 0.45 |
| LIG3 | **15.95 (13.35 - 19.66)** | **15.39 (14.53 - 21.30)** | 0.47 | 0.51 |
| MDC1 | **14.93 (12.95 - 18.08)** | **15.95 (14.73 - 26.40)** | 0.29 | 0.31 |
|  |  |  |  |  |
| SHLD1 | **14.04 (12.62 - 17.65)** | **17.88 (16.08 - 25.18)** | 0.008 | 0.01 |
| TRIP13 | **18.08 (15.95 - 25.18)** | **14.73 (13.78 - 16.60)** | 0.008 | 0.01 |

**Table S2.** Median overall survival of GBM patients from The Cancer Genome Atlas GBM dataset. Patients receiving TMZ were stratified by median expression of the indicated gene and survival was determined by analysis in cBioPortal. p-values are from Log rank test and q-values are corrected for multiple comparisons with Benjamini Hochberg procedure. 95% confidence intervals are displayed in parentheses.

**References**

1. Akagawa, R. *et al.* UBC13-Mediated Ubiquitin Signaling Promotes Removal of Blocking Adducts from DNA Double-Strand Breaks. *iScience* **23**, 101027 (2020).

2. Piett, C. G., Pecen, T. J., Laverty, D. J. & Nagel, Z. D. Large-scale preparation of fluorescence multiplex host cell reactivation (FM-HCR) reporters. *Nat Protoc* **16**, 4265–4298 (2021).
